# More than one quarter of Africa’s tree cover is found outside areas previously classified as forest

**DOI:** 10.1038/s41467-023-37880-4

**Published:** 2023-05-02

**Authors:** Florian Reiner, Martin Brandt, Xiaoye Tong, David Skole, Ankit Kariryaa, Philippe Ciais, Andrew Davies, Pierre Hiernaux, Jérôme Chave, Maurice Mugabowindekwe, Christian Igel, Stefan Oehmcke, Fabian Gieseke, Sizhuo Li, Siyu Liu, Sassan Saatchi, Peter Boucher, Jenia Singh, Simon Taugourdeau, Morgane Dendoncker, Xiao-Peng Song, Ole Mertz, Compton J. Tucker, Rasmus Fensholt

**Affiliations:** 1 Department of Geosciences and Natural Resource Management, University of Copenhagen, Copenhagen, Denmark; 2 Global Observatory for Ecosystem Services, Department of Forestry, Michigan State University, East Lansing, MI 48823 USA; 3 Department of Computer Science, University of Copenhagen, Copenhagen, Denmark; 4 Laboratoire des Sciences du Climat et de l’Environnement, CEA/CNRS/UVSQ/Université Paris Saclay, Gif-sur-Yvette, France; 5 Department of Organismic and Evolutionary Biology, Harvard University, Cambridge, MA 02138 USA; 6 Pastoralisme Conseil, Caylus, France; 7 Laboratoire Evolution et Diversité Biologique, CNRS, UPS, IRD, Université Paul Sabatier, Toulouse, France; 8 Department of Information Systems, University of Münster, Münster, Germany; 9 Université Paris Saclay, Gif-sur-Yvette, France; 10 Jet Propulsion Laboratory, California Institute of Technology, Pasadena, CA 91109 USA; 11 UMR SELMET, CIRAD, Université Montpellier, Montpellier, France; 12 Earth and Life Institute, Environmental Sciences, Université catholique de Louvain, Louvain-la-Neuve, Belgium; 13 Department of Geographical Sciences, University of Maryland, College Park, MD 20740 USA; 14 Earth Sciences Division, NASA Goddard Space Flight Center, Greenbelt, MD 20771 USA

**Keywords:** Biogeography, Forestry, Forest ecology

## Abstract

The consistent monitoring of trees both inside and outside of forests is key to sustainable land management. Current monitoring systems either ignore trees outside forests or are too expensive to be applied consistently across countries on a repeated basis. Here we use the PlanetScope nanosatellite constellation, which delivers global very high-resolution daily imagery, to map both forest and non-forest tree cover for continental Africa using images from a single year. Our prototype map of 2019 (RMSE = 9.57%, bias = −6.9%). demonstrates that a precise assessment of all tree-based ecosystems is possible at continental scale, and reveals that 29% of tree cover is found outside areas previously classified as tree cover in state-of-the-art maps, such as in croplands and grassland. Such accurate mapping of tree cover down to the level of individual trees and consistent among countries has the potential to redefine land use impacts in non-forest landscapes, move beyond the need for forest definitions, and build the basis for natural climate solutions and tree-related studies.

## Abstract (web summary)

Recent analyses have suggested that tree cover in non-forest ecosystems may be much higher than expected. Here, the authors map tree cover down to the individual tree level for the entire continent of Africa and find that almost 30% is found outside areas classified as forests.

## Introduction

Forests and other tree-based ecosystems contribute to the removal of CO_2_ emissions, and are thus central to climate change mitigation strategies aiming to achieve net zero CO_2_ emissions targets^1^. Accordingly, the Glasgow Pact from COP26, to which more than 100 countries are signatory, stresses the importance of halting and reversing global deforestation by 2030^2^. In order to achieve success, actions to halt deforestation and forest degradation require the direct support from high-quality monitoring systems that deliver measurement, reporting, and verification (MRV) of forest area and change consistently and comparably among countries^3^. However, tree losses do not only occur in dense high-carbon forests, but also in landscapes of scattered trees that do not form closed-canopy forests. Conversely, tree gains in these non-forest landscapes are often not perceived positively as they can contribute to destabilizing open ecosystems^4^.

The United Nations Food and Agriculture Organization (FAO) provides relatively clear definitions for forests, but regroups remaining landscapes with trees into “other wooded land” and “other land”^5^. These categories include a variety of tree-based systems, among them savannahs and woodlands, shrub- and bushlands, trees on agricultural land, and clustered trees in woodlots. The physical boundaries separating forest and non-forest are relatively clear in the Northern Hemisphere. However, many African landscapes are drylands, where trees outside forests are the major form of woody vegetation. Previous studies have found that, following the FAO definition, forests cover only 21.4% of Africa, with an additional 14.9% considered as “other wooded land”^6^. The remaining 63.7% is classified as “other land”, which also includes agricultural plantations, agroforesty, urban trees, and a wide variety of tree complexes outside forests in agricultural lands. These trees outside forests play a vital role in ecological stability, local economies, livelihoods, and food security^7,8^.

The quantitative assessment of trees in both forested and non-forested landscapes is crucial to reducing emissions from deforestation and forest degradation, as well as increasing sequestration through forest restoration, agroforestry, and other restoration interventions. Quantifying trees outside forests would also enable assessment of woody encroachment and the identification of areas threatened by increasing woody cover. However, ambiguous definitions, unclear MRV techniques that may differ from country to country, and scarce technical and financial resources in many developing countries limit the reliability and credibility of current approaches^3,9–11^. Moreover, defining better classes to separate large trees, with high ecological and economic value, from shrubs would be an important improvement over current definitions. Similarly, for closed canopies the classification of forests by canopy height is desirable, both to ensure that small shrubs are not mapped as tree cover, and to differentiate between primary, secondary, or plantation forests. This requires the incorporation of canopy height datasets, such as satellite, aeroplane-, or UAV-based lidar measurements. Overall, the principal problem remains the consistent assessment of all tree and forest resources, across both countries and years.

Satellite data of moderate spatial resolution (10–30 m) are the prevailing data source for mapping and monitoring tree cover change at continental-to-global scales^12^. However, the 10–30 m resolution does not allow the characterization of individual trees outside forests, although there have been attempts to map pixel fractional cover with methods such as spectral unmixing^13,14^. Recent studies have shown that very high spatial resolution (0.5 m) satellite data and state-of-the-art machine learning techniques are able to map individual trees across large areas^15,16^, which is one important requirement for accurate reporting schemes. Previous work used images from many commercial satellites, resulting in a temporal mixing of data across more than a decade^16^. However, the use of data from different years and dates is problematic, not only because tree change information is obscured, but also because trees are not consistently mapped due to variations in phenology. Moreover, extending high-resolution tree mapping to continental or global scales is limited by technical challenges in data processing and storage. Furthermore, sub-metre imagery is prohibitively expensive for most non-governmental organizations to acquire and process data over large study areas. Finally, the lack of temporally consistent imagery makes it difficult to monitor fine-scale tree cover change caused by tree plantations, agroforestry, selective logging, deforestation, or woody encroachment. Overall, it is currently difficult to implement consistent assessments at continental scale and across years based on sub-metre satellite data, in spite of their high value. Therefore, a critical gap remains in the repeated mapping of forest and non-forest trees at high resolution in a consistent temporal window and at a continental level.

Here, we address these limitations by using high-resolution satellite imagery from a nano-satellite constellation, freely available for the tropics via Norway’s International Climate and Forest Initiative (NICFI) programme^17^. Our major objective is to map all forest and non-forest trees at continental scale across Africa, and at a precision exceeding all previous attempts to map woody vegetation across large scales. We use a machine learning approach to segment tree canopy cover in 3 m PlanetScope satellite imagery across Africa, down to the level of individual scattered trees. We quantify the contribution of trees outside forests to total tree cover per country, and find that at the continental scale, 29% of all tree cover is found outside areas classified as forest in a current state-of-the-art map based on Sentinel-2 10 m imagery^18^.

## Results

### A very high-resolution map of African tree cover

**Fig. 1:**
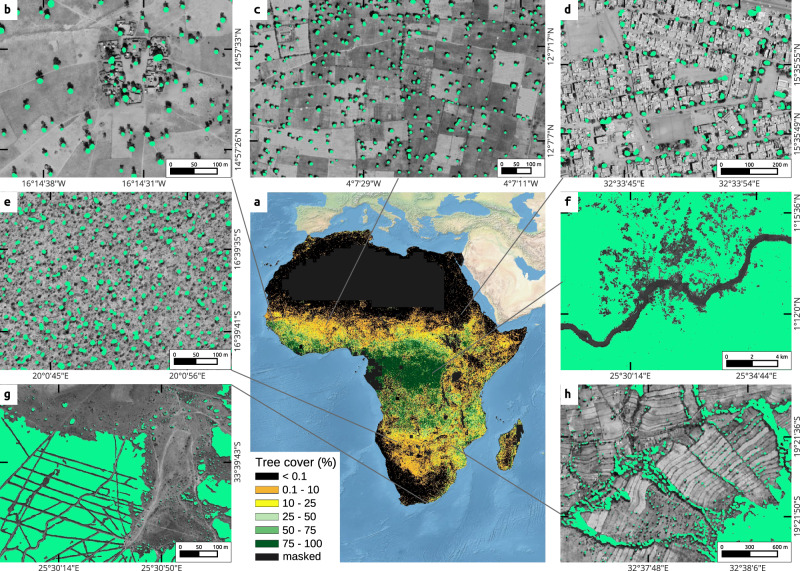
Mapped tree cover across areas of different tree densities. **a** Percentage tree cover, at 1 km spatial resolution; **b**–**h** examples of predicted tree cover overlaid on Google Maps satellite imagery (Imagery © 2022 CNES / Airbus, Landsat / Copernicus, Maxar Technologies, Map data ©2022), in: **b** a village in Senegal; **c** agricultural fields in Burkina Faso; **d** an urban environment in Khartoum, Sudan; **e** Miombo woodlands in Angola; **f** deforestation in the Democratic Republic of Congo (DRC); **g** *Eucalyptus* plantations in South Africa; **h** terrace farming in Zimbabwe. The ocean basemaps in **a** are from www.naturalearth.com.

We used 3 m resolution satellite imagery from Planet Labs Inc to generate composites covering continental Africa in 2019. The raw images were provided by the PlanetScope constellation of nanosatellites, with 4-band scenes available globally at daily temporal resolution^19^. We organized and mosaiced more than 230,000 satellite scenes in a grid of 1 × 1° tiles. The time window for each tile was inferred from the green vegetation phenology of the area (see Methods), such that tree leaf cover is maximized while grasses have passed their productivity peak. The availability of daily PlanetScope images was key to generating cloud-free images for a narrow time frame of a few weeks within a single year. In cloud-prone regions, the time window was extended progressively up to several months (see Methods). We then used a deep learning model trained with about 130,000 manual training samples to segment tree crown cover for all of Africa at 1 m resolution. We only labelled trees or groups of trees that were clearly identifiable as a woody plant with an associated shadow, which typically excludes bushes and shrubs below about 5 m (see Methods). We upsampled the images from 3 to 1 m to increase the model prediction performance by preserving the high quality of the manually delineated training samples (Supplementary Fig. 1). In croplands, savannahs and low-density woodlands, trees were mapped as individual crowns. Semi-dense and closed forests were mapped as closed canopies, with no separation of individual crowns (Fig. 1).

To derive percent tree cover from the binary tree/no-tree map, we aggregated our results into 30 × 30 m grid cells. These cells were first grouped into non-forest areas with a maximum tree cover of 10% and 25% from our map, two widely used forest definition thresholds^5,20^. Then, if canopy cover exceeded 25%, we considered the cell to be a forest, which we further grouped into different canopy heights using a spaceborne LiDAR based map from a previous study^21^. Note that our results are not constrained by these definitions and, given the level of detail in the mapping, full flexibility exists for selecting thresholds and resolutions to apply, or to use different canopy height maps^22^ (Supplementary Fig. 2a, b).

**Fig. 2:**
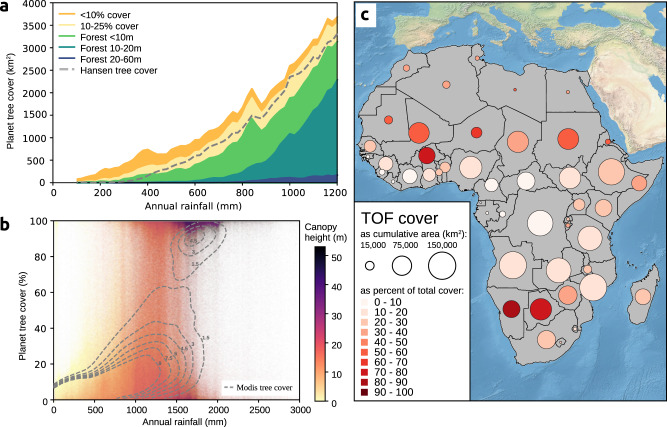
Distribution of tree cover by rainfall and percent cover. **a** Total tree cover area by rainfall. Tree cover is classified into forest at different heights^21^, and into two groups of trees outside forest (TOF) with canopy cover <10% and 10–25%, respectively. A current state-of-the-art global map is added for comparison^12^. This figure highlights the regions below 1200 mm rainfall and the full map is shown in Supplementary Fig. 2a. **b** Tree cover vs. rainfall at 100 m resolution using a random sample of 10 million grids, with hue as the forest height^21^, and isolines overlaid for MODIS tree cover^49^ from 100 000 samples at 250 m, with isoline units as relative probability per rainfall and cover grid cell. **c** Contribution of trees outside forests (TOF) to total tree cover at country scale. We group trees in 30 × 30 m grids and define a cell as non-forest if the canopy cover is below 25%, and as forest, if it exceeded 25%. Tree cover is subsequently accumulated for each country. The ocean basemaps are from www.naturalearth.com.

To analyze the distribution of trees across different climatic zones, we summed tree cover by annual rainfall^23^ (Fig. 2a, Supplementary Fig. 2c). A previously published and widely used global tree cover map was added for comparison purposes^12^. We find that, at continental scale, the PlanetScope tree cover matches the global map for forests and even for areas of 10–25% tree cover, especially in higher rainfall areas, but the global map completely misses tree cover below 10%, which is the dominant form of tree cover in low rainfall areas. Across Africa, we show that 15.8% of tree cover is located in areas with less than 25% canopy cover, and 6.0% of the tree cover is in areas with less than 10% canopy cover. A total of 0.16% of tree cover is in hyper-arid areas (0–150 mm rainfall), 0.7% in arid areas (150–300 mm rainfall), 4.3% in semi-arid areas (300–600 mm), 27.5% in sub-humid areas (600–1200 mm), and 67.0% in humid areas (>1200 mm). Interestingly, a random sample of tree cover plotted against rainfall shows a homogeneous distribution of tree cover along the rainfall gradient without a gap at 60–80% tree cover (Fig. 2b), which is observed in the widely used MODIS tree cover and has previously been interpreted as a sign of alternative stable ecosystem states^24^. The lack of such a gap in our tree cover map thus supports the argument by Hanan et al. that this was an artefact from the statistical processing of the MODIS tree cover map^25^.

### Distribution of trees outside forests

We compared the total tree cover from non-forest areas (here defined as <25% tree cover) against the total tree cover for 45 African countries. Even though trees in non-forest areas contribute only a minor part to the total tree cover at continental scale (Fig. 2a, Supplementary Fig. 2a), at national level trees outside forests constitute more than 50% of all tree cover in nine countries (Fig. 2c), namely Botswana, Burkina Faso, Eritrea, Libya, Mali, Namibia, Niger, Mauritania, and Sudan. This implies that previous moderate-resolution tree cover maps are of little use to quantify national woody resources for these countries.

**Fig. 3:**
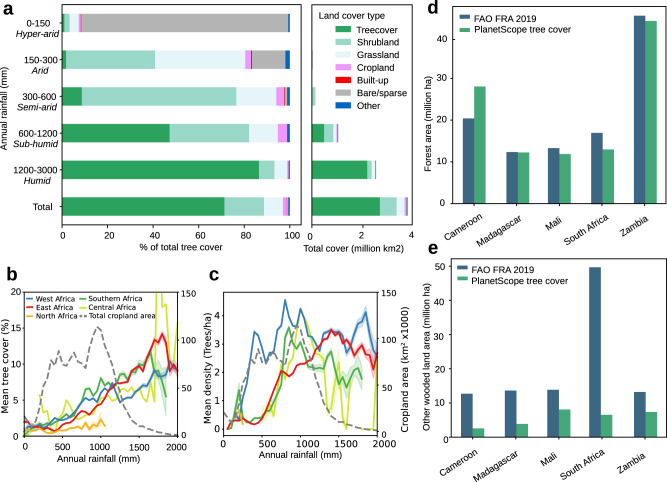
Tree cover by forest type and land cover. **a** Distribution of mapped tree cover by land cover type and precipitation zone, with land cover classes from the Worldcover product^18^. See Supplementary Table 1 for mean tree cover by land cover. **b** Distribution of non-forest trees on African croplands, showing mean tree cover and **c** mean tree density on croplands. The right-side y-axis is the total cropland area, and the shaded background of the lines represent the 95% confidence interval. See Supplementary Fig. 3 for grasslands. **d** Reported tree cover compared to FAO statistics for ‘Forest’, defined as areas with >10% tree cover and **e** ‘other wooded land’ defined as areas with 5–10% tree cover, or >10% cover of trees, bushes, and shrubs. For more countries see Supplementary Fig. 4.

The very high resolution of our tree cover map was then used to quantify the proportion of tree cover found across land cover classes, by leveraging the 10 m resolution WorldCover land cover map^18^. Overall, across all rainfall regions, we find that 28.7% of tree cover is found outside of land classified as ‘tree cover’ according to WorldCover (Fig. 3a). Additionally we show that for dryland regions (up to 1200 mm rainfall), the majority of tree cover is found in areas classified as shrublands, grasslands and deserts (‘bare/sparse’ category), with 90.9% of tree cover found in bare/sparse land cover for hyper-arid areas, 39.6% in grasslands and 39.0% in shrublands for arid areas, 67.9% in shrublands for semi-arid areas, and 34.9% in shrublands for sub-humid areas (Fig. 3a).

At continental scale, while a relatively large amount of tree cover (~29%) is outside the ‘tree cover’ land cover class, only 1.7% of tree cover is found in croplands. However, for local communities in African drylands, many of the primary uses of cropland trees are food, fodder, shade, and fuel-wood, or their role in agroforestry systems^8^. Such cropland trees are usually widely spaced and can be individualized in our tree cover map. This lends itself to the analysis of the number, density, crown size, and distribution of trees on croplands. We assumed that all segmented objects on croplands are individual tree crowns, with a correction applied where objects with crown size >200 m^2^ are assumed to be tree clusters and considered as multiple trees of 200 m^2^. We found a total number of about 433 million trees on African croplands, with a mean tree density of 2.55 (±5.81) trees per hectare.

Dividing Africa into northern, western, eastern, central, and southern Africa (Supplementary Fig. 5a), we find that western Africa has the highest mean density of cropland trees across most of the rainfall gradient, although this pattern is not replicated in the mean tree cover (Fig. 3b, c). This suggests large numbers of smaller trees on western African croplands, especially in the rainfall regions of 300–1200 mm. As annual rainfall increases, there are different rainfall thresholds for each region at which cropland tree density suddenly rises, at about 250 mm for western Africa, 500 mm for eastern Africa, and 700 mm for central and southern Africa. A possible explanation may lie in the greater use for agroforestry of drought resistant Sahelian trees in western Africa, compared to eastern and southern Africa.

### Comparison with FAO statistics and canopy height maps

We compared our numbers of area cover with the FAO statistics reported by each country. Here we group our results following the FAO definitions of “forest” and “other wooded land”, where “forest” is defined as areas >0.5 ha, with tree height above 5 m and a canopy cover of more than 10%; and “other wooded land” is defined as areas >0.5 ha, with tree height above 5 m and a canopy cover of 5–10%, or a combined cover of trees and shrubs above 10% (Fig. 3d, e). The category “other land with tree cover” was not considered because no statistics exist for most of the countries. Forest areas matched relatively well with our results for most countries: at continental scale, we map 7,519,197 km² as forest and the FAO reports 6,374,249 km^2^ for the year 2019. Interestingly, our results tend to map more areas as forest compared to the FAO statistics, which could be a result of different definitions of “forest”. For example, FAO “forest” includes forestry plantations but not tree crop plantations, but both are included in our map when mature. We map markedly fewer areas as “other wooded land” than reported by the FAO (1,830,553 km² as compared to 4,438,199 km^2^), likely because bushes and small shrubs are not included in our map.

**Fig. 4:**
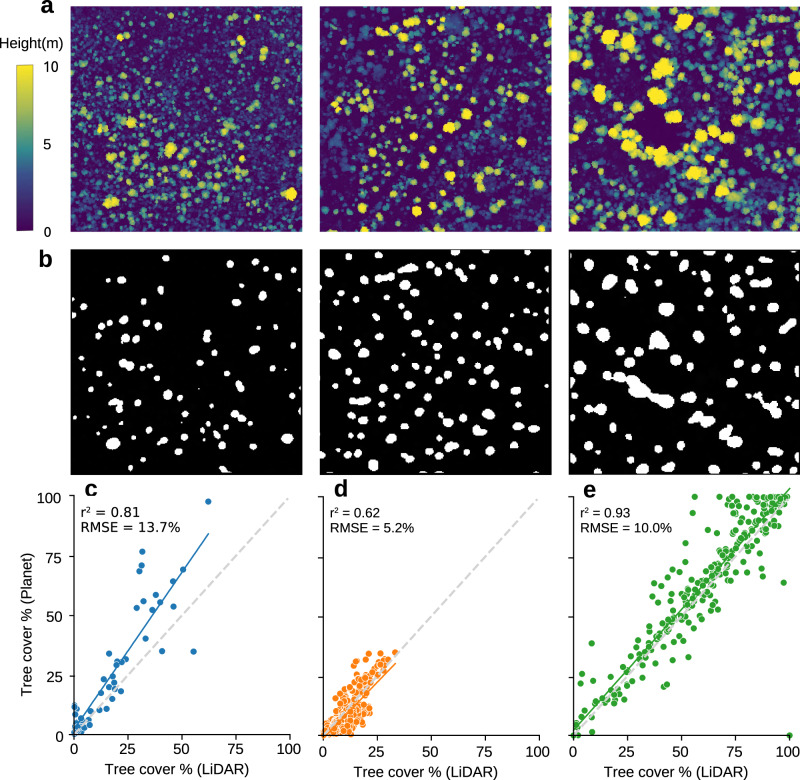
Validation of tree segmentation results with canopy height models. **a** Airborne LiDAR canopy height models for three scenes in Karingani Game Reserve, Mozambique. **b** Corresponding binary tree cover predictions from PlanetScope map. **c**–**e** Scatter plot between PlanetScope tree cover and CHM tree cover if a minimum tree height of 5–6 m is used for the CHM data (see Supplementary Fig. 6a for different height thresholds); for **c** 59 sample plots in Senegal, ranging from 2–15 ha size (5 m height threshold), **d** 400 random plots in Mozambique, each 50 ha size (6 m height threshold), **e** 400 random plots in DRC, each 50 ha size (6 m height threshold).

When comparing our tree cover statistics with those of the FAO, it is thus important to consider the tree height at which our method starts to map trees, as the FAO also includes shorter shrubland with a cover >10% in “other wooded land”. Here, we compared our results with canopy height models (CHM) from airborne LiDAR and UAV stereo photogrammetry from Senegal, DRC and Mozambique, and show that we typically do not map bushes, small shrubs and trees below 5–6 m (Fig. 4, Supplementary Fig. 6). Furthermore, a comparison with 178,750 isolated trees derived from sub-metre resolution optical imagery from the Sahel^16^ shows that isolated tree crowns above 30 m² were reliably mapped (less than 20% missed), while 44.2% of the crowns below this threshold were omitted (Supplementary Fig. 7). Therefore, our maps may be used to redefine the category of “other wooded land” by separating single scattered trees in areas of low tree cover from shrubs and bushes.

## Discussion

This study reports on a comprehensive, systematic, very high-resolution accounting of all tree-systems, not only forests, for a single year across Africa. This achievement marks a milestone towards the monitoring of woody resources, and has profound implications for biomass monitoring, conservation biology, landscape ecology, and sustainable forestry, among others^26^. In particular, this approach brings together several important considerations relevant to MRV tools needed to mobilize actions for natural climate solutions (NCS). An accurate assessment of tree cover at metric resolution for both forests and non-forest areas over large areas at a high temporal frequency has the potential to redefine our knowledge of land use impacts on tree cover change, carbon stocks, emissions, and removals. Ultimately, if tree cover is reported from the aggregation of data at single tree level, this level of accuracy and precision would allow the variable “tree cover” to be used in policy-making as an instrument for improved and transparent management of tree resources.

The inclusion of non-forest trees also highlights the challenge of a clear separation between forest and non-forest trees. Applying the FAO definition (<10% cover for 0.5 ha) on our map, the number of trees outside forests is low (5.2% of the total cover). This is however because most of the trees we find would qualify as “forest” according to this definition. If comparing with a previous state-of-the-art map based on 10-m Sentinel-2 data^18^, we find that 29% of tree cover is found outside the areas previously classified as tree cover. A continentally consistent high resolution tree cover product is thus an important step to comparing and harmonizing the different forest definitions used across countries and institutions.

As part of this study, tools were developed to automate the process of generating uniform mosaics of PlanetScope scenes for any grid tile globally, and for predicting tree cover for a specific year or date range. Given that PlanetScope imagery is available every day for the whole world, these processing methods lay the groundwork for global tree cover mapping at annual scale and very high spatial resolution. While this would require further annotation data to cover new ecosystems, the labelling effort is expected to be lower due to the variety of ecosystems already trained for the Africa model. Additionally, the free availability of NICFI PlanetScope data for the tropics makes this methodology feasible for developing countries at national level.

Looking ahead, the same method could potentially be used as an important component of a global tree cover monitoring programme. Such a framework would dramatically enhance our capability to detect and map tree cover change at a high temporal frequency, extending existing deforestation monitoring systems to the point where the removal of single trees and thickets is tracked. With increased spatial resolution and fidelity of canopies and individual tree crowns, it also increases the ability to measure changes within forests, especially forest degradation, which, for some regions, is becoming as important in terms of area and carbon as deforestation^27^. The ability to include the full range of tree cover densities lays a foundation for a globally harmonized approach to monitoring ongoing ecosystem restoration projects, such as those prioritized under the “UN decade of ecosystem restoration”, among others. In addition to the current international efforts to reduce deforestation and forest degradation^28,29^, this extended monitoring capability would provide critical support to sustainable land management, community-based forest and natural resources management, and national forest inventory and assessment programmes. Furthermore, tree monitoring could also be applied to open ecosystems^30^, where increases in tree cover through woody encroachment^31^ or mishandled afforestation programmes^32^ can be detrimental to ecosystem functioning and biodiversity^33^. Identifying and monitoring increasing tree cover in such ecosystems could alert managers and policy makers to potential biome shifts that could be detrimental for biodiversity and pastoralists.

Currently, one caveat in scaling up this method to a global scale is the vast quantity of high quality manually delineated training labels that would be required to ensure consistent predictions across the numerous and diverse local vegetation types globally. Alternative ways to produce reliable training data are an ongoing topic of research^34^, and it is likely that the automatic integration of auxiliary very high-resolution data such as UAV imagery or LiDAR will play an increasingly important role in creating training data. Furthermore, the map provided here is a prototype and there are several limitations in the use of PlanetScope data that require future research. First, the detection of small, isolated trees with crown sizes below 30 m^2^ is challenging, which hinders the applicability of our approach to monitoring newly planted trees, natural regeneration, and bushes. Second, only isolated trees can be reliably identified as individuals. We also need to explore the feasibility of splitting closed-canopy predictions into single trees, which would improve the estimation of carbon stocks by enabling the use of allometric models based on single tree crowns^16,35^. Third, current tree height information is not available at a high spatial resolution, so the applied separation of forest types is based on the relatively coarse GEDI LiDAR data. Moreover, while comparisons with LiDAR data showed that we can roughly state that trees and shrubs above 5 m are detected, this number should not be considered as a hard threshold but more as an orientation. It cannot always be guaranteed that bushes and smaller shrubs are not partially mapped, and also there is no strict guarantee that all trees above 5 m are captured. Future versions of this map may extend the use of LiDAR data to training the model, leaving less uncertainty regarding the definition of the mapped trees. Fourth, from the original spatial resolution of the images, it is not possible to detect gaps between dense tree crowns and forest canopies, leading to overestimations in some forests and shrublands. This effect is aggravated by understory that fills gaps between larger trees, for example in woodlands. Fifth, PlanetScope images are not available before 2015. The possibility of using our data to calibrate Landsat time series to assess changes over longer times needs to be explored.

Tree cover maps generated from current nanosatellites do not have the level of precision of sensors that record images at sub-metre resolution, so there is a trade-off between precision and cost, scalability, and repeatability, although ongoing improvements to the current product will reduce many of the above-mentioned limitations. Our prototype maps represent a combination of continental scale and single tree precision that sets the stage for a new era of global tree cover mapping that has the potential to overcome forest definition ambiguity. Consequently, this study provides a more robust and complete evaluation of tree cover, bringing in the full Agriculture, Forestry, and Other Land Use (AFoLU) domain of landscapes. It also enables landscape-oriented approaches to climate change mitigation while also supporting adaptation that integrates livelihoods. Third, it provides monitoring for forest landscape restoration and ecosystem degradation, but is not limited to forests, and, fourth, it underpins sustainable land management and land degradation assessments. Ultimately, our work provides an opportunity to integrate and serve the critical needs of many major conventions, including the UNFCCC, UNCBD, and UNCCD^36–38^, all of which are challenged by a diversity of national forest definitions which, at least in terms of CO_2_ emission and sequestration accounting, will become less problematic if the focus is on individual trees.

## Methods

### Overview

We created custom mosaics of very high resolution satellite imagery covering the African continent in 2019, with imagery dates selected for optimal visibility of tree crowns. We then used deep learning techniques to train a model that can segment tree crown cover in the imagery, and applied this model to the entire dataset to produce a continental scale map of tree cover. The full processing workflow is shown in Supplementary Fig. 8.

### Satellite images

We used high-resolution satellite images from the PlanetScope constellation of nanosatellites, with all data obtained from Planet Labs through a research license. The images consist of 4-band multispectral scenes at 3 m resolution, with atmospherically corrected surface reflectance values for the Blue, Red, Green, and Near-infra-red bands, and were available via the Planet API with the PSScene *analytic_sr* product bundle^39^. For the continental study area, we downloaded about 230,000 individual PlanetScope scenes from 2019, covering a total area of 24,222,164 km^2^.

### Mosaic generation

To organize the large data volumes, we divided the study area into a grid of 1 × 1 degree tiles and generated a custom mosaic of PlanetScope scenes for each tile. The creation of custom mosaics instead of available Planet basemap imagery was pivotal, as the provided basemaps are designed to maximize visualization by combining scenes from many different days, which limits the consistent detection of tree crowns, especially in drylands with strong seasonal vegetation differences. Instead, we developed an automated algorithm to download and mosaic Planet scenes based on the local phenological conditions of each grid tile. For best visibility of tree cover, a date range was selected where both evergreen and deciduous trees were in full foliage, but interfering signal from tall grassy vegetation was minimized, such as at the beginning of the dry season. We used the MODIS/Terra phenology product^40^ to determine the local mean days for senescence, mid-greendown, and dormancy thresholds. We also included an indicator if the majority of the tile was dominated by deciduous or evergreen vegetation; this information was derived from the Copernicus Dynamic Land Cover map^41^. The date range of included scenes for a given mosaic tile was then taken as senescence to mid-greendown for deciduous tiles, and mid-greendown to dormancy for non-deciduous tiles (Supplementary Fig. 9c). This ensured that the trees in deciduous areas had not yet started leaf shedding, and that grass interference in evergreen areas was minimized. After filtering by date range, we then used a dynamic scene-placing algorithm to select scenes and partial scenes until the entire mosaic tile was filled. The criteria used to select scenes include the date and instrument type, as well as metadata properties on quality such as the amount of clouds, haze, shadow, ‘clear confidence’, and a general quality indicator. For tiles where there were not enough scenes due to frequent cloud cover, the date range was automatically progressively extended forwards and backwards in time until a coverage threshold of 99% was achieved. We did not perform any temporal merging of the 3 m PlanetScope scenes to create composite images, because small differences in view angle and orthorectification of subsequent acquisitions introduce significant noise at the scale of small trees.

The selected scenes per tile were clipped to their partial footprints, downloaded in parallel, reprojected to WGS84, and merged into a mosaic. To reduce the sharp edges between scenes, a histogram matching algorithm was then applied using Landsat reference images^42^, with the reference images chosen from the same date range as the mosaic grid cell. This procedure matched the varied individual scene surface reflectances to the consistent histogram distribution of the much larger Landsat scenes, resulting in a uniform final mosaic (Supplementary Fig. 9a, b). The software tools developed for the mosaic generation process were fully automated, such that with a single input of a study area analysis ready custom mosaics can be generated anywhere in the world.

### Mapping canopy cover with deep learning

We used a custom deep learning framework developed in Python to segment the tree crown cover in PlanetScope images. This framework is an extension of the UNet architecture described in Brandt et al.^16^. The UNet is a convolutional neural network (CNN) architecture originally developed for medical segmentation tasks, but has proven to be highly suited for tree crown segmentation^16,35,43,44^. We used an adapted UNet model with batch normalization and self-attention, and the model was trained with a batch size of 8 and a patch size of 512 × 512. To enhance the training data, image augmentation was performed with multiple transformations including flipping, cropping, affine transformations, and linear contrast enhancement. The loss function used during training was an adapted version of the focal Tversky loss^45,46^, which is designed to handle imbalanced classes and allows careful tuning between omission and commission.

The UNet model was trained using more than 130,000 manually labelled training samples from two persons, including both individual trees and merged canopy clusters. A tree was only labelled if it could be clearly identified as a woody plant with an associated shadow in Google Earth or Bing maps, if this was not the case, the area was excluded from the training process. The labelling process was done in two iterations: the first round used random areas covering different ecosystems (such as croplands, savannas, shrublands, woodlands, forest) over all Africa, as seen in Supplementary Fig. 5b. We then trained a model and predicted tree cover over the continent. Subsequently, we conducted a visual inspection and thereafter a second round of labelling in areas where the model did not perform well. The final samples were distributed across 21 countries, covering various forest types, rainfall conditions, and local ecosystems throughout western, eastern, and southern Africa (Supplementary Fig. 5b). One change from the previously published framework^16^ was to incorporate a dynamic resampling functionality into the deep learning pipeline, to allow for on-the-fly in-memory upsampling of the source imagery from 3 m to 1 m during both training and prediction. The reason for upsampling the PlanetScope images was to improve the prediction quality by preserving the high fidelity of the manual training data. The training labels were polygons with many vertices at sub-pixel resolution, and this detail would be lost when rasterizing them to 3 m. By rasterizing the labels to 1 m, and training with upsampled 1 m data, the model can instead use contextual clues such as shadows to produce sub-pixel predictions at 1 m. We found a large difference between predicting at 3 m and predicting on the upsampled 1 m images, with the 1 m images resulting in significantly more small trees detected, and finer detail on large crowns (Supplementary Fig. 1).

Due to the large amount of training data and the many different situations across the continental study area, it was found that the model would sometimes confuse different types of vegetation, which appeared the same at the local patch level. For example, a small patch of uniformly dense *Eucalyptus* plantation in South Africa may look nearly the same in infra-red as a patch of dense cropland in Senegal, thus degrading the model by training with opposing labels. This problem was solved by training two separate models, one focusing on forest areas, and one specializing on non-forest trees. It was found that for the forest model the upsampling step was not necessary, as there is less additional benefit of predicting at 1 m vs 3 m, compared to the advantage of increased context from a larger effective patch area at 3 m. Thus the final architecture is an ensemble prediction of the forest model at 3 m, and the non-forest model at 1 m.

### Evaluation of tree cover results with LiDAR data

To evaluate the accuracy of the model’s predictions of tree cover, we compared our binary tree cover map with canopy height maps (CHMs) derived from aerial LiDAR and stereo UAV imagery. We did not use manual labels because these are not independent and are impacted by the choice of the person doing the labelling. The LiDAR and UAV stereo photography data cover different landscape types, such as savannah, shrubland, woodland, rainforest, and cropland, and were available for sample sites in Senegal (3 cm and 70 cm resolution; 4.6 km^2^ area), DRC (1 × 1 m; 79.6 km^2^ area), and Mozambique (1 × 1 m; 68.5 km^2^ area). Using the resulting CHM images, we sampled 400 random plots of 50 ha each for DRC and Mozambique and used the 59 sample plots of 2–15 ha from Senegal. We then compared the percent tree cover per plot with PlanetScope tree cover over the same area. Because the CHM data are based on tree height, a certain minimum height had to be chosen as a cutoff to define a tree crown, in order to compare to tree cover at the plot level. We used different LiDAR minimum height thresholds ranging from 3 m to 7 m. While there were country-specific variations, the best correlation with the CHM was found with a 5–6 m threshold, indicating that the PlanetScope tree cover maps trees above approximately 5-6 m (Supplementary Fig. 6a). Using a 6 m threshold, we then obtained an *R*^2^ = 0.81 and RMSE = 16.2% for Senegal, *R*^2^ = 0.62 and RMSE = 5.2% for Mozambique, and *R*^2^ = 0.93 and RMSE = 10.0% for DRC. The overall RMSE across all countries was 9.19% for a height threshold of 6 m, and 9.57% with a threshold of 5 m.

To determine the bias of our tree cover relative to the LiDAR data, we calculated the bias in tree cover both at plot level and overall across all plots. Here we use the relative biases, or systematic relative error, which are defined as:

1 $${{{{{{\rm{bias}}}}}}}_{{{{{\rm{plot}}}}}}=\frac{1}{N}\mathop{\sum }\limits_{i=1}^{N}\frac{({Y}_{{{{{{\rm{obs}}}}}}}-{Y}_{{{{{{\rm{pred}}}}}}})}{{Y}_{{{{{{\rm{obs}}}}}}}}\times 100$$

2 $${{{{{{\rm{bias}}}}}}}_{{{{{{\rm{total}}}}}}}=\frac{\mathop{\sum }\nolimits_{i=1}^{N}({Y}_{{{{{{\rm{obs}}}}}}}-{Y}_{{p{{{{\rm{red}}}}}}})}{\mathop{\sum }\nolimits_{i=1}^{N}{Y}_{{{{{{\rm{obs}}}}}}}}\times 100$$

For a LiDAR height threshold of 5 m we obtain a plot bias of 1.29% (overestimation) and total bias of −6.90% (underestimation). The difference in plot vs total bias suggests that areas with high cover such as in DRC are underpredicted, contributing to a negative total bias, while across all plots the bias is slightly positive. Supplementary Fig. 6b shows the biases for varying height thresholds from 3 m to 7 m, with the lowest biases corresponding to a height of 5–6 m.

### Evaluation of single tree prediction results compared to sub-metre imagery

In forest or dense thickets, the PlanetScope tree cover did not split the canopy into individual crowns, but for scattered trees the crowns were mapped as individuals. However, compared to satellite sensors with sub-meter resolution, the 3 m sensor resolution of PlanetScope imagery excluded the ability to detect very small isolated trees <10 m² crown size, and lowered prediction accuracy for smaller crown sizes <30 m². To determine the number of isolated trees thus missed, we compared our results to the predictions on 50 cm WorldView imagery performed in a previous study^16^.

However, Brandt et al. used images from many different years and phenological periods, which were not uniformly orthorectified, causing considerable challenges in directly comparing the two maps. To limit the effect of these differences, we selected 50 cm sample scenes from their dataset that were from later than 2015, in a similar month as the PlanetScope mosaic, and where automatic image alignment was successful. Furthermore, we restricted the scenes to croplands, as the PlanetScope model does not map trees as individuals if tree cover is dense. We then compared the number of 50 cm mapped trees that were detected by the PlanetScope model, where a 50 cm tree is considered successfully detected when it overlaps with any PlanetScope tree, to account for occasional clustering of trees in the PlanetScope model (Supplementary Fig. 7). Note that Supplementary Fig. 7a shows a ‘best-case’ scenario of an area in Nigeria, where the Planet imagery is clear and there is minimal shift. It can be concluded that detections become reliable at 30 m² crown size or larger, for smaller crown sizes, about half of the trees are missed by our model.

### Environmental data and auxiliary datasets

We used land cover data from both the ESA WorldCover 2020 product at 10 m^18^ and the Global Land Cover and Land Use Change product at 30 m^47^ to mask out water bodies at prediction time. The WorldCover map was also used to mask croplands for the analysis of trees on croplands.

We used historical rainfall data from the Climate Hazards Group Infra-red Precipitation with Station (CHIRPS) product to generate a reference rainfall map^23^. We averaged the annual rainfall data from 1982 to 2018 and bilinearly interpolated to 100 m for analysis with tree cover.

We further used tree cover from Hansen et al.^12^, where the tree cover for 2019 was produced by starting with the tree cover in 2000 and adding the available annual forest gain up to 2013, then subtracting annual forest losses up to 2019.

Finally, we used forest canopy height data from both Lang et al.^21^ at 10 m and Potapov et al.^22^ at 30 m to group our tree cover into height classes (Fig. 2a, b, Supplementary Fig. 2a).

We did not merge the two products, but performed our analysis against both to highlight the effect of different canopy height maps on the resulting classes.

### Reporting summary

Further information on research design is available in the Nature Portfolio Reporting Summary linked to this article.

## Supplementary information

Supplementary Information

Reporting Summary

## Data Availability

Planetscope imagery was partly from a departmental research license and partly from Norway’s International Climate and Forest Initiative (NICFI) satellite data Level 2 programme. NICFI Planetscope imagery in tropical areas is available for non-commercial purposes from Planet Labs at https://www.planet.com/nicfi/. However, we did not use the basemaps provided in the frame of the NICFI programme but generated our own mosaics from the raw data. The derived tree cover maps produced in this study have been deposited in the Zenodo database and are available at 10.5281/zenodo.7764460^48^. Note that the maps are under active development and improved versions will be released in the future. The ESA WorldCover product^18^ used for the land cover analyses is available for download at 10.5281/zenodo.5571936. The global canopy height data from Lang et al.^21^ are available for download at https://share.phys.ethz.ch/~pf/nlangdata/ETH_GlobalCanopyHeight_10m_2020_version1/. The global canopy height data from Potapov et al.^22^ are available for download at https://glad.umd.edu/dataset/gedi. The global tree cover data from Hansen et al.^12^ are available for download at https://storage.googleapis.com/earthenginepartners-hansen/GFC-2021-v1.9/download.html.

## Code availability

The code for the tree detection framework based on U-Net is publicly available at 10.5281/zenodo.3978185. Support and more information are available from A.K. (ak@di.ku.dk or ankit.ky@gmail.com) or F.R. (flr@ign.ku.dk). Code for preparation of imagery from PlanetScope raw scenes has been deposited in the Zenodo database and is available at 10.5281/zenodo.7764360. Support and more information are available from F.R. (flr@ign.ku.dk). The python libraries used in the image preparation and prediction pipelines are publicly available and include GDAL v3.1.2, rasterio v1.2, tensorflow v2.5, geopandas v0.9, and the Planet python API v1.4.7.
