# Supplementary material for: More than one quarter of Africa’s tree cover is found outside areas previously classified as forest
Source: Nat Commun. 2023 May 2;14:2258. doi: 10.1038/s41467-023-37880-4 (PMC10154416; doi:10.1038/s41467-023-37880-4)
Supplement: Supplementary file 1 — Supplementary Information [file 41467_2023_37880_MOESM1_ESM.pdf]

Supplementary Information to:

More than one quarter of Africa's tree cover is found outside areas previously classified as forest

Florian Reiner[1]*, Martin Brandt[1]*, Xiaoye Tong[1], David Skole[2], Ankit Kariryaa[1,3], Philippe Ciais[4], Andrew Davies[5], Pierre Hiernaux[6], Jérôme Chave[7], Maurice Mugabowindekwe[1], Christian Igel[3], Stefan Oehmcke[1,3], Fabian Gieseke[3,8], Sizhuo Li[1,9], Siyu Liu[1], Sassan Saatchi[10], Peter Boucher[5], Jenia Singh[5], Simon Taugourdeau[11], Morgane Dendoncker[12], Xiao-Peng Song[13], Ole Mertz[1], Compton J. Tucker[14], Rasmus Fensholt[1]

1) Department of Geosciences and Natural Resource Management, University of Copenhagen, Copenhagen, Denmark
2) Global Observatory for Ecosystem Services, Department of Forestry, Michigan State University, East Lansing, MI 48823, USA
3) Department of Computer Science, University of Copenhagen, Copenhagen, Denmark
4) Laboratoire des Sciences du Climat et de l'Environnement, CEA/CNRS/UVSQ/Université Paris Saclay, Gif-sur-Yvette, France
5) Department of Organismic and Evolutionary Biology, Harvard University, Cambridge, MA 02138, USA
6) Pastoralisme Conseil, Caylus, France
7) Laboratoire Evolution et Diversité Biologique, CNRS, UPS, IRD, Université Paul Sabatier, Toulouse, France
8) Department of Information Systems, University of Münster, Münster, Germany
9) Université Paris Saclay, Gif-sur-Yvette, France
10) Jet Propulsion Laboratory, California Institute of Technology, Pasadena, CA 91109, USA
11) UMR SELMET, CIRAD, Université Montpellier, Montpellier, France
12) Earth and Life Institute, Environmental Sciences, Université catholique de Louvain, Louvain-la-Neuve, Belgium
13) Department of Geographical Sciences, University of Maryland, College Park, MD 20740, USA
14) Earth Sciences Division, NASA Goddard Space Flight Center, Greenbelt, MD 20771, USA

* Correspondence to flr@ign.ku.dk or mabr@ign.ku.dk

Supplementary Table 1 | Mean tree cover by land cover class.
Landcover classes are derived from WorldCover 2020 [1]

Land cover type (WorldCover)	Mean tree cover (%)	Standard deviation
Tree cover	65.47	30.01
Shrubland	14.79	17.28
Grassland	6.43	11.04
Cropland	4.14	5.99
Built-up	7.01	8.18
Bare/Sparse	0.50	3.28

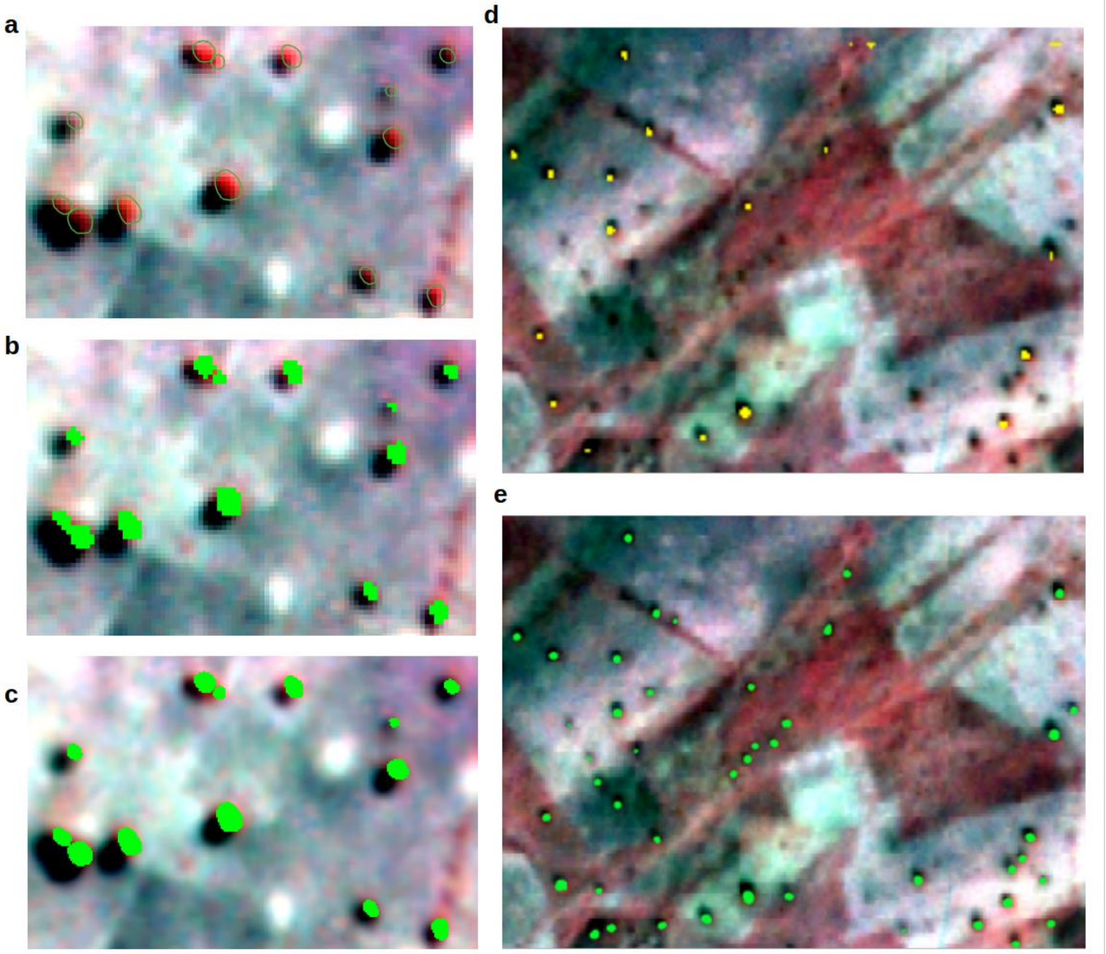

Supplementary Fig. 1 | Improved scattered tree predictions after upsampling to 1 m. To preserve higher detail of the polygon annotations during training, the 3 m raster images were upsampled to 1 m using bilinear interpolation, and the annotations rasterized to 1 m. This resulted in a higher level of detail in the output tree crown segmentations, and improved predictions of small scattered trees for the upsample. **a**, Original 3 m image with hand delineated polygon annotations. **b,** 3 m image with annotations rasterised at 3 m. **c,** Upsampled 1 m image with annotations rasterised to 1 m. **d**, Predictions with 3 m model. **e**, Predictions with images and annotations upsampled to 1 m, but otherwise same training data and settings as the 3 m model. All predictions are shown overlaid on PlanetScope satellite imagery (Imagery © 2019 Planet Labs Inc. All use subject to the Participant License Agreement).

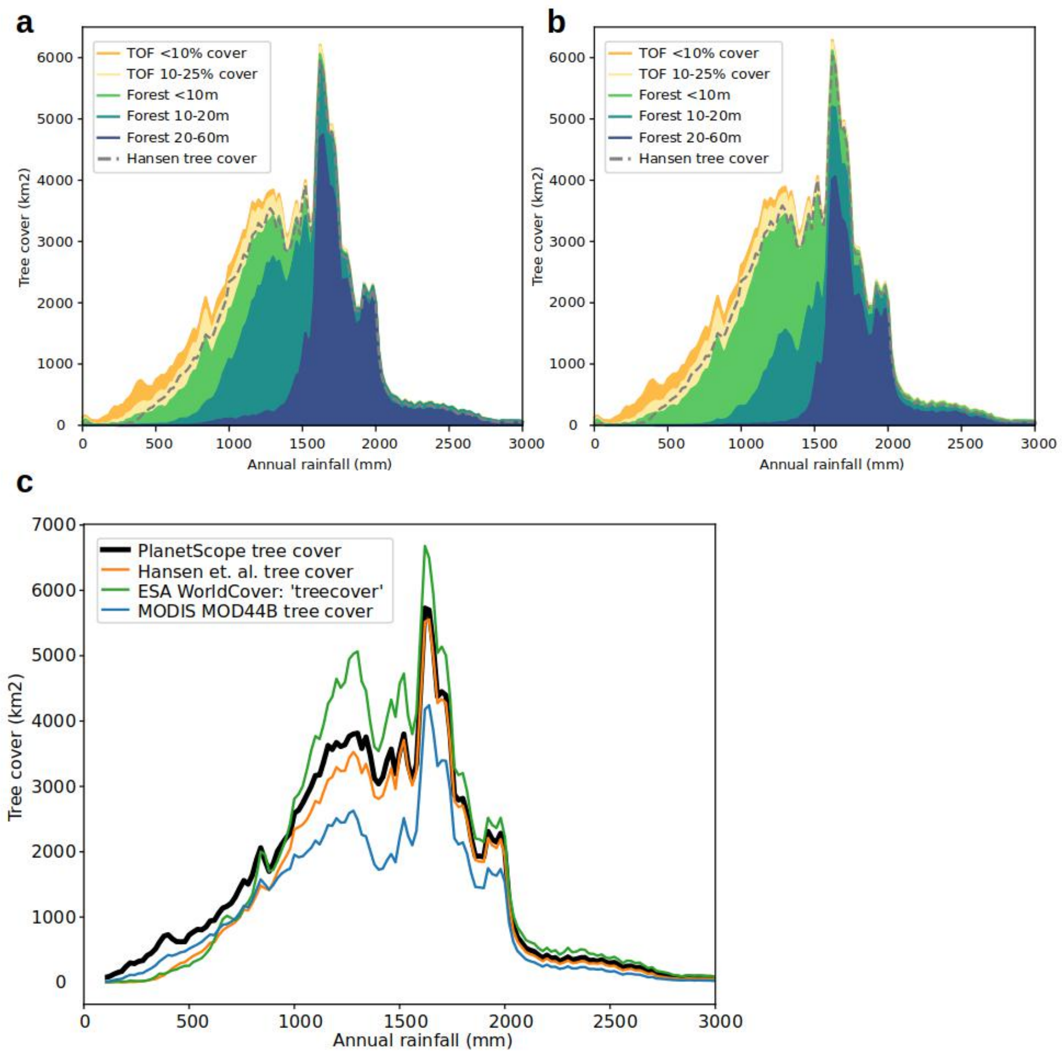

Supplementary Fig. 2 | Tree cover by rainfall.

a, Tree cover by rainfall and canopy height, with cover grouped into height classes using canopy height from Lang et al. [2], and **b,** using canopy height from Potapov et al. [3]. **c,** Comparison of summed cover by rainfall for different tree cover products, and the 'treecover' class of the WorldCover land cover map [1].

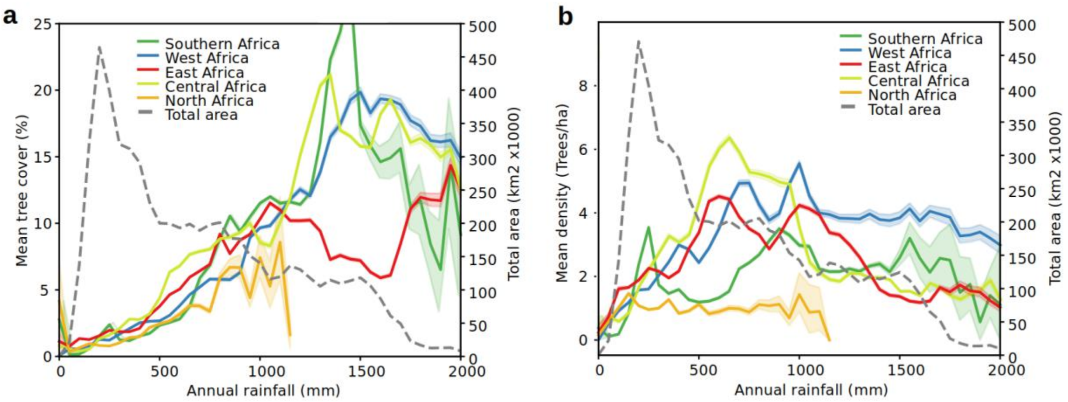

Supplementary Fig. 3 | Distribution of non-forest trees on grasslands by region. a, Mean tree cover and **b,** mean tree density (trees per ha), on land classified as 'grassland' in the ESA WorldCover 2020 product [1]. The right-side y-axis is the total grassland area, and the shaded background of the lines represents the 95% confidence interval.

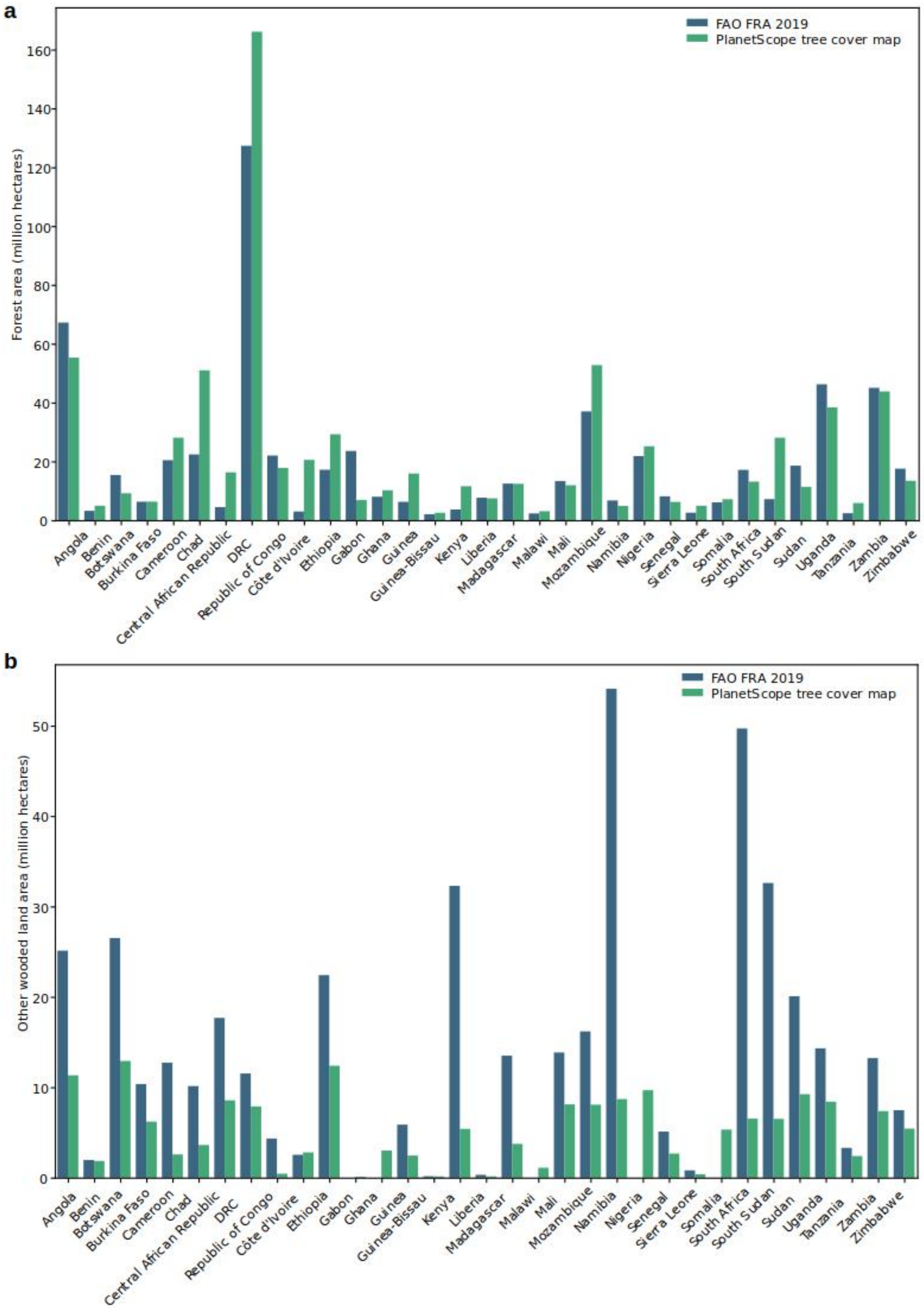

Supplementary Fig. 4 | Reported tree cover compared to FAO statistics for, a, the “Forest” category, defined by the FAO as areas >0.5 ha with >10% tree cover and more than 5 m height, and **b,** the “Other wooded land” category, defined by the FAO as areas >0.5 ha with 5-10% tree cover of more than 5 m height, or >10% combined cover of trees, bushes and shrubs.

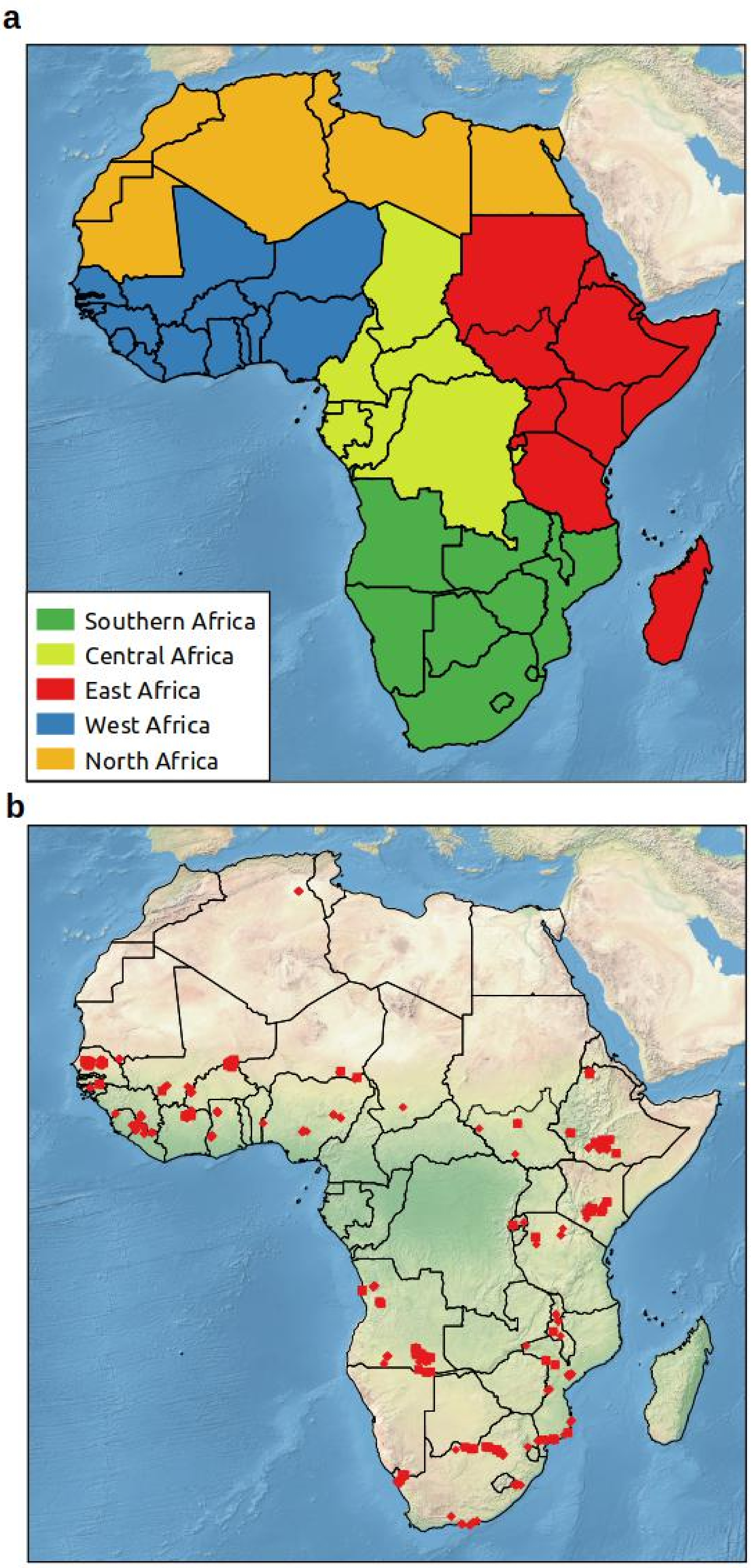

Supplementary Fig. 5 | African regions and training data distribution.
a, African regions as defined by the African Union. **b,** The distribution of annotated training data samples. The ocean basemaps are from www.naturalearth.com

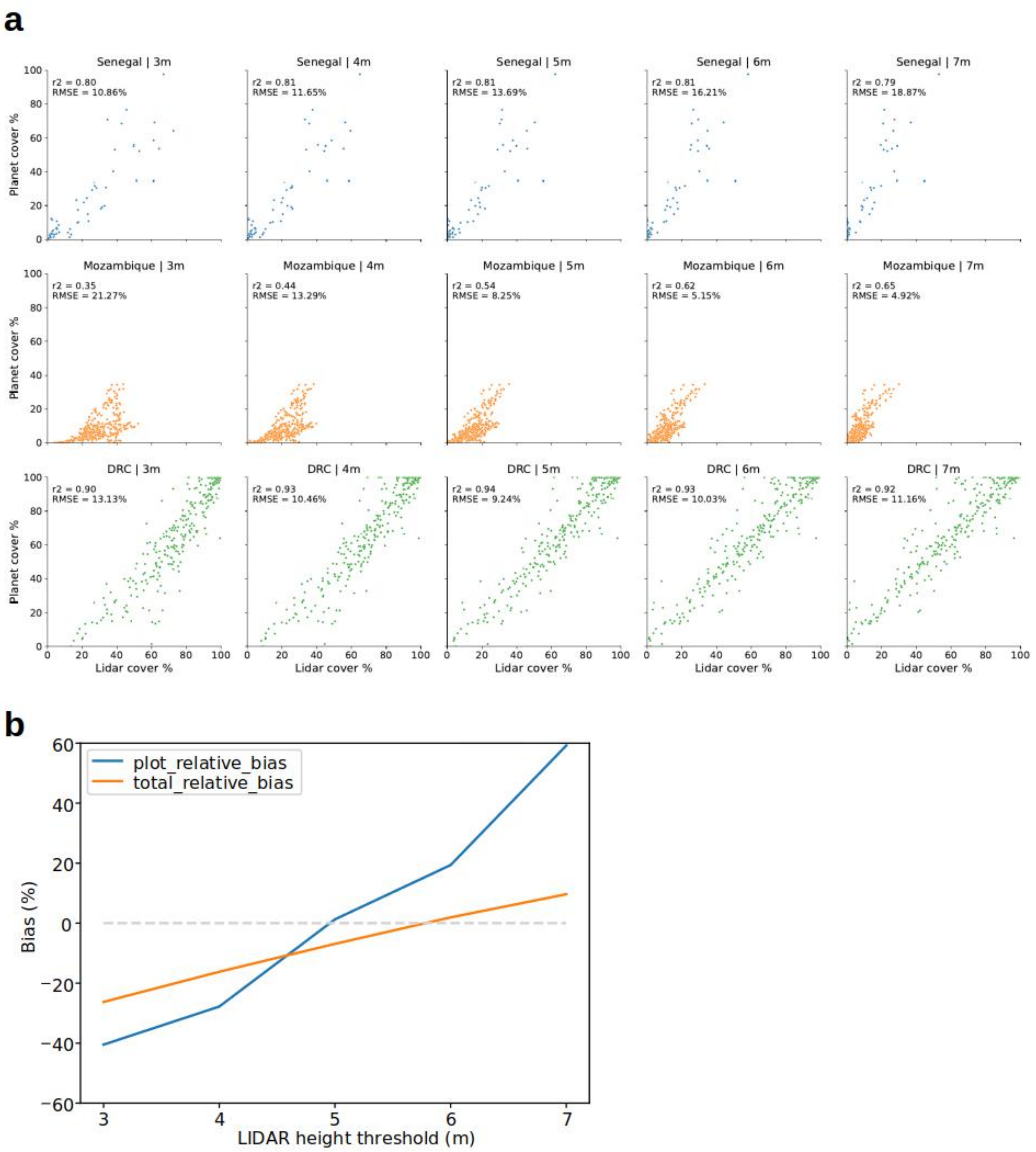

Supplementary Fig. 6 | Comparison of mapped percent cover with canopy height data. **a,** Comparison of our mapped tree cover in percent to the tree cover from aerial canopy height models, by country and height threshold. The height threshold refers to the minimum height used as a cutoff to segment the canopy height map to binary tree cover, thus a higher threshold results in lower percent cover from the canopy height map. Shown are scatter plots from 59 sample plots of 2-15 ha for Senegal, 400 random 50 ha plots in Mozambique, and 400 random 50 ha plots in DRC. **b,** The plot relative bias and total relative bias of our mapped cover vs the reference canopy height data, across the range of different height thresholds.

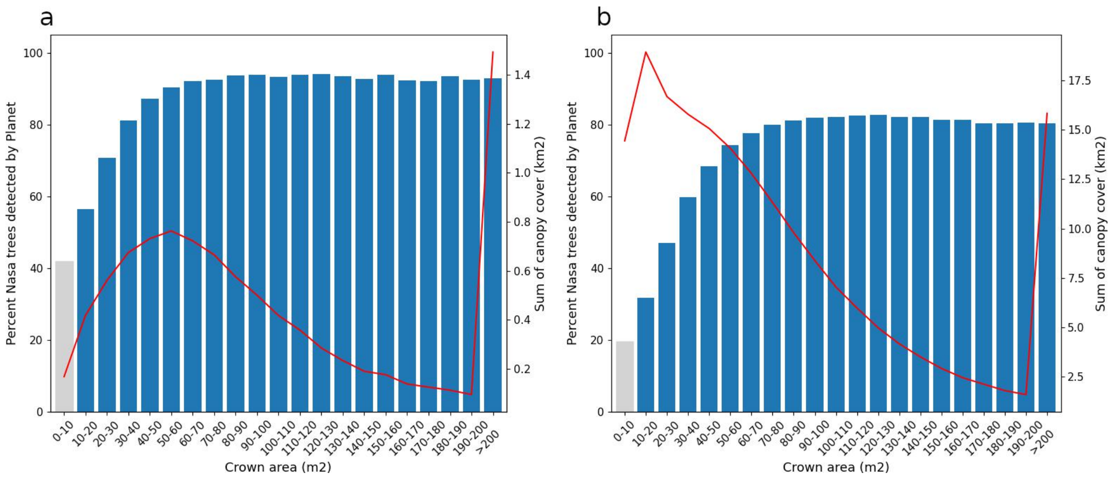

Supplementary Fig. 7 | Evaluation of tree detection. These graphs show how much percent of the trees were detected by the PlanetScope model, using trees from Brandt at al. 2020 [4], using **a,** a sample of 178,750 trees for a clear, well-aligned scene, **b,** a sample of 6,239,787 trees across Sahelian croplands. The sum of total canopy cover by crown size area is shown as a red line on the right axis.

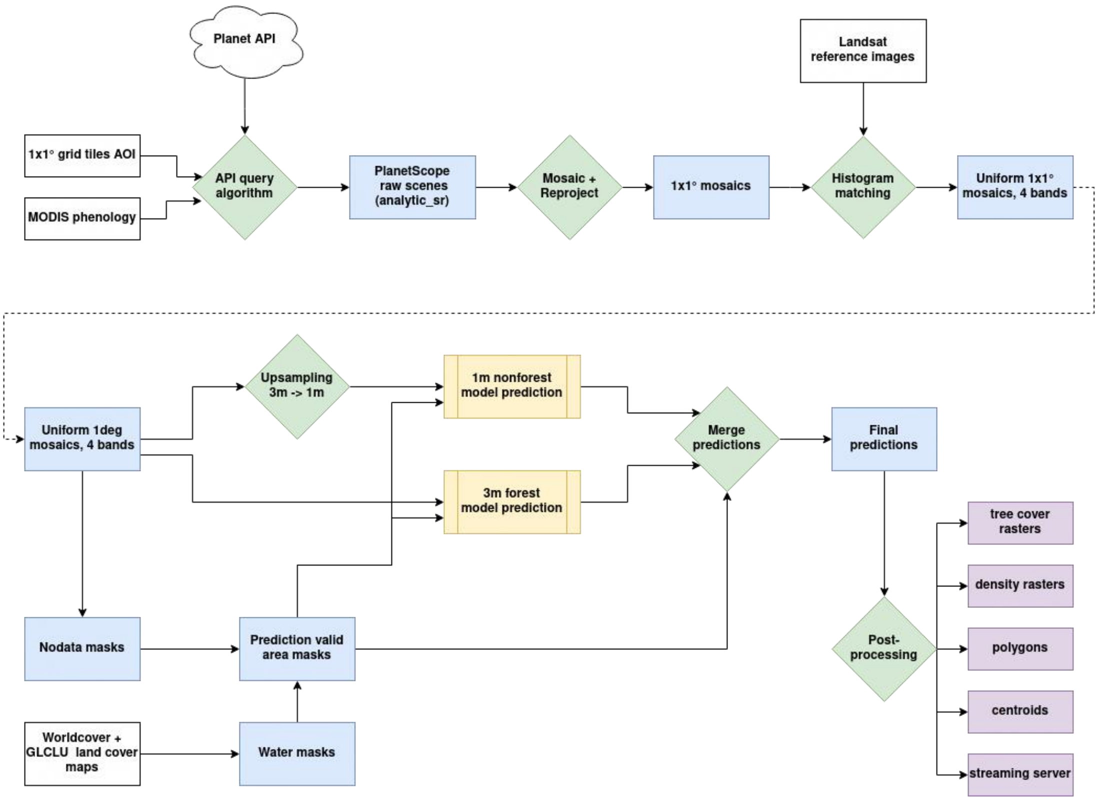

Supplementary Fig. 8 | Processing workflow for mosaic generation and tree prediction.

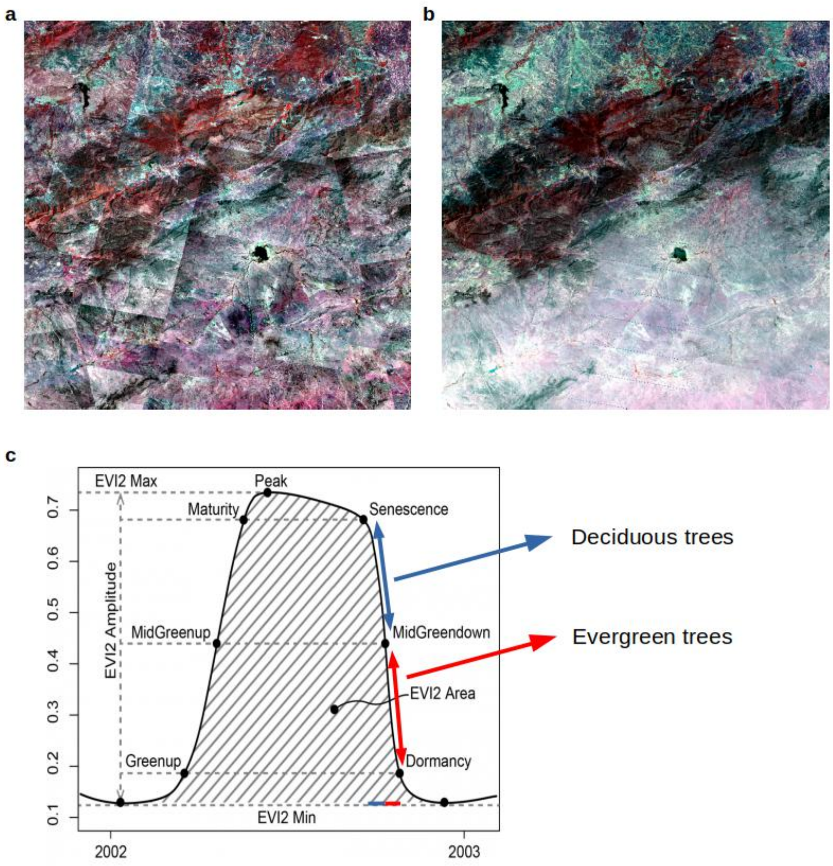

Supplementary Fig. 9 | Mosaic creation. a, Mosaic scenes before histogram matching. **b,** Final mosaic after histogram matching with Landsat reference images [5]. **c,** Illustration of phenology window for scene selection, overlaid on a MODIS phenology diagram from the USA-NPN [6].

Supplementary References:

1. Zanaga, D. *et al.* ESA WorldCover 10 m 2020 v100. (2021) doi:10.5281/ZENODO.5571936.
2. Hansen, M. C., Stehman, S. V. & Potapov, P. V. Quantification of global gross forest cover loss. *Proc. Natl. Acad. Sci. U. S. A.* **107**, 8650–8655 (2010).
3. Potapov, P. et al. Mapping global forest canopy height through integration of GEDI and Landsat data. *Remote Sens. Environ.* **253**, 112165 (2021).
4. Brandt, M. et al. An unexpectedly large count of trees in the West African Sahara and Sahel. *Nature* **5563**, (2020).
5. U.S. Geological Survey, Landsat 8 Level 2, Collection 2, Tier 1, available at: https://developers.google.com/earth-engine/datasets/catalog/LANDSAT_LC08_C02_T1_L2 (accessed May 2022)
6. USA National Phenology Network. 2019. Land Surface Phenology 2001-2017 for the United States. USA-NPN, Tucson, Arizona, USA, available at www.usanpn.org/data/land_surface_phenology. (accessed June 2022)
